# Outcome of kidney function after ischaemic and zero-ischaemic laparoscopic and open nephron-sparing surgery for renal cell cancer

**DOI:** 10.1186/s12882-019-1215-3

**Published:** 2019-02-04

**Authors:** Jan Ebbing, Felix Menzel, Paolo Frumento, Kurt Miller, Bernhard Ralla, Tom Florian Fuller, Jonas Busch, Justin William Collins, Christofer Adding, Hans Helge Seifert, Peter Ardelt, Christian Wetterauer, Timm Westhoff, Carsten Kempkensteffen

**Affiliations:** 1 University Hospital Basel, Urological University Clinic Basel-Liestal, Spitalstrasse 21, 4051 Basel, Switzerland; 2 Department of Urology, Charité - University Hospital, Berlin, Germany; 3 Karolinska Institutet, Unit of Biostatistics, Institute of Environmental Medicine (IMM), Stockholm, Sweden; 4 Department of Molecular Medicine and Surgery (MMK), Karolinska Institutet, Stockholm, Sweden; 5 Marien Hospital Herne – University Clinic of the Ruhr-University Bochum, Medical Clinic I, Herne, Germany; 6 Department of Urology, Karolinska - University Hospital, Solna, Stockholm, Sweden; 7 Department of Urology, Franziskus Hospital Berlin, Berlin, Germany

**Keywords:** Partial nephrectomy, Nephron-sparing surgery, Kidney function, Acute kidney injury, AKI, Chronic kidney disease, CKD, Ischaemia time, Zero ischaemia

## Abstract

**Background:**

Nephron-sparing surgery (NSS) remains gold standard for the treatment of localised renal cell cancer (RCC), even in case of a normal contralateral kidney. Compared to radical nephrectomy, kidney failure and cardiovascular events are less frequent with NSS. However, the effects of different surgical approaches and of zero ischaemia on the postoperative reduction in renal function remain controversial.

We aimed to investigate the relative short- and long-term changes in estimated glomerular filtration rate (eGFR) after ischaemic or zero-ischaemic open (ONSS) and laparoscopic NSS (LNSS) for RCC, and to analyse prognostic factors for postoperative acute kidney injury (AKI) and chronic kidney disease (CKD) stage ≥3.

**Methods:**

Data of 444 patients (211 LNSS, 233 ONSS), including 57 zero-ischaemic cases, were retrospectively analysed. Multiple regression models were used to predict relative changes in renal function. Natural cubic splines were used to demonstrate the association between ischaemia time (IT) and relative changes in renal function.

**Results:**

IT was identified as significant risk factor for short-term relative changes in eGFR (ß = − 0.27) and development of AKI (OR, 1.02), but no effect was found on long-term relative changes in eGFR. Natural cubic splines revealed that IT had a greater effect on patients with baseline eGFR categories ≥G3 concerning short-term decrease in renal function and development of AKI. Unlike LNSS, ONSS was significantly associated with short-term decrease in renal function (ß = − 13.48) and development of AKI (OR, 3.87). Tumour diameter was associated with long-term decrease in renal function (ß = − 1.76), whereas baseline eGFR was a prognostic factor for both short- (ß = − 0.20) and long-term (ß = − 0.29) relative changes in eGFR and the development of CKD stage ≥3 (OR, 0.89).

**Conclusions:**

IT is a significant risk factor for AKI. The short-term effect of IT is not always linear, and the impact also depends on baseline eGFR. Unlike LNSS, ONSS is associated with the development of AKI. Our findings are helpful for surgical planning, and suggest either the application of a clampless NSS technique or at least the shortest possible IT to reduce the risk of short-time impairment of the renal function, which might prevent AKI, particularly regarding patients with baseline eGFR category ≥G3.

## Background

The gold standard of care for managing renal cell cancer (RCC) remains surgical tumour excision [1, 2]. Currently, nephron-sparing surgery (NSS) is recommended for clinically localised tumours (cT1–2) by the European Association of Urology (EAU) and the American Urological Association (AUA) guidelines [1, 2]. Outside of specialised centres, the standard surgical procedure is open partial nephrectomy. However, an interest in the implementation of minimally invasive NSS has continuously increased [3, 4]. Several contemporary studies demonstrated oncological outcomes of patients undergoing laparoscopic NSS (LNSS) that were equivalent to those of patients undergoing open NSS (ONSS) [5–7].

Compared to radical nephrectomy, kidney failure and cardiovascular events are less frequent with NSS [2, 8–12]. However, whether NSS also improves overall survival remains controversial [11, 13–20]. Results from the only randomized trial of NSS versus radical nephrectomy (EORTC-30904), which included mainly patients with a normal baseline estimated glomerular filtration rate (eGRF) and a normal contralateral kidney, revealed that the impact of NSS on eGFR did not result in improved non-cancer related mortality and less cardiovascular events in general [11, 16, 17]. In contrast, other recent retrospective data showed that in patients who had chronic kidney disease (CKD) before surgery, lower postoperative eGFR was associated with increased mortality, independently of age and comorbidities [18], and that there is an increased risk of death from any cause or cardiovascular death with decreased postoperative renal function, but the latter study was not adjusted for the baseline renal function [19]. These results suggest that the surgery-related factors that influence the non-oncological outcome measures are much less important than internistic disorders such as diabetes, arterial hypertension, or medical CKD [11, 16, 18]. Just recently, a relationship between renal function and cancer-specific mortality was also debated [21]. However, several studies have shown a significantly increased risk of progression of renal failure, cardiovascular disease, and subsequent mortality in patients developing CKD [22–24], and approximately 16–40% of patients treated with NSS develop postoperative CKD stage ≥3 [25, 26], which is defined as an eGFR < 60 mL/min/1.73 m^2^ by the Kidney Disease: Improving Global Outcomes (KDIGO) criteria of kidney disease [27].

Reductions in renal function mainly related to renal parenchymal mass loss, renal ischaemia, and reduced baseline kidney function are seen in NSS patients, but the effects of different surgical approaches (e.g., ONSS and LNSS), and of zero ischaemia (ZI) in this aspect remain controversial. Due to the known oncological equivalence of LNSS versus ONSS for localised RCC, many are interested in understanding surgery-related factors influencing non-oncological outcome measures.

Therefore, the purpose of this work was to demonstrate the dynamics of renal function after NSS, to identify risk factors for the development of acute kidney injury (AKI) and CKD stage ≥3 after NSS, and to compare the effects of ONSS and LNSS for RCC as well as ZI (no renal clamping during surgery) on postoperative renal function.

## Methods

We used our prospectively populated database and archive to retrospectively identify 444 patients who were treated with NSS for RCC at the Department of Urology of the Charité–University Hospital between 1999 and 2010. A total of 211 patients were treated with LNSS and 233 patients were treated with ONSS. Exclusion criteria were metastatic RCC, recurrent RCC, bilateral RCC, and preoperative end-stage chronic kidney disease (CKD stage 5/kidney failure). Furthermore, angiomyolipoma, oncocytoma, and other non-malignant tumours were not included in order to limit this series to only pure primary RCC patients.

This study was approved by the local ethics committee of the Charité–University Hospital Berlin. Data collection was performed in accordance with the requirements of the local ethics committee. All patients provided written informed consent.

Patient demographics, tumour characteristics, surgical characteristics, and preoperative and postoperative renal function were obtained.

Warm renal ischaemia was performed according to the surgeon’s decision. Arterial vessel clamping for hilar control was performed using bulldog clips for LNSS and Satinsky clamps or vessel loops (tourniquets) for ONSS. Tumour resection, management of the tumour ground, and renal parenchyma/collecting system reconstruction were similarly performed during LNSS and ONSS. Tumours were resected or enucleated whenever possible using standard diathermy resection techniques. Large vessels were oversewn with absorbable sutures or controlled with titanium ligating clips. The renal parenchyma defect was closed using an absorbable hemostat patch fixed by gathering sutures of the parenchyma or by using a hemostatic matrix. An injured urinary collection system was sutured and a ureteral stent was inserted when indicated at the surgeon’s discretion.

To monitor renal function, baseline serum creatinine was measured preoperatively (time 0), during the hospitalisation course (times A and B), and during variable postoperative follow-up times (times C, D, and E) according to oncological follow-up investigations. Data were retrospectively collected for the highest serum creatinine value during the planned hospital stay (time A), serum creatinine prior to discharge from hospital (time B), approximately 6 weeks (time C) and 12 months (time D) after surgery, and at the latest recorded follow-up time (time E). The median measurement time was 1 day (interquartile range [IQR], 1–2) postoperatively for time A, 4 days (IQR, 2–6) postoperatively for time B, 47 days (IQR, 30–105) postoperatively for time C, 13 months (IQR, 12–15) postoperatively for time D, and 50 months (IQR, 35–81) postoperatively for time E. Glomerular filtration rate (GFR) was estimated based on the Modification of Diet in Renal Disease (MDRD) formula [28]. Estimated GFR (eGFR) was calculated to investigate the absolute and relative (%) changes in renal function between time 0 and postoperative times A–E in the overall NSS cohort (NSS-C) and in the following subgroups: NSS group with intraoperative renal ischaemia (NSS-RI), NSS group without intraoperative renal ischaemia (NSS-NRI), LNSS group with intraoperative renal ischaemia (LNSS-RI), ONSS group with intraoperative renal ischaemia (ONSS-RI), NSS group with development of postoperative AKI (NSS-AKI), NSS group without development of postoperative AKI (NSS-NAKI), NSS group with baseline eGFR category G1 (NSS-G1), NSS group with baseline eGFR category G2 (NSS-G2), and NSS group with baseline eGFR category ≥G3 (NSS ≥ G3).

Kidney function was staged based on eGFR categories G1–G5 according to the Kidney Disease: Improving Global Outcomes (KDIGO) criteria [27] at baseline and all follow-up times. CKD stage ≥3 was defined by eGFR < 60 mL/min/1.73 m^2^ (eGFR category ≥G3) independent of markers of renal damage [27]. An increase in serum creatinine levels of at least ≥50% or ≥ 0.3 mg/dL within 48 h postoperatively was considered AKI [29].

Statistical analyses were completed using SPSS Statistics version 23.0 (IBM Corp., Armonk, NY, USA) and R (http://www.r-project.org). Continuous variables were summarised by medians with IQR, whereas sample proportions were used to describe categorical and binary outcomes. To compare distributions, we used the Mann-Whitney U test (2 groups) or Kruskal-Wallis test (more than 2 groups) for continuous responses; we used Pearson’s chi-squared test for categorical responses. Friedman’s test was used to assess absolute and relative (%) changes in eGFR over time (measurement times A–E vs. baseline).

We used multiple linear and logistic regression models to predict the short-term relative changes in eGFR from baseline at time A (model 1) and the long-term relative changes in time D (model 2), and to identify predictors of AKI (model 3) and CKD stage ≥3 (eGFR < 60 mL/min/1.73 m^2^) at time D (model 4). We implemented two different versions of each model: (a) one including ischaemia time (IT) linearly, and (b) one using categorical variables (ZI vs. ischaemia in general or 21–30 min of ischaemia [reference] vs. different ITs of 0 min [ZI], 1–10 min, 11–20 min, > 30 min) as well as the interaction between ZI and eGFR categories (G1 [reference] vs. G2 or vs. ≥G3).

Natural cubic splines were used to demonstrate the association between IT and relative change in eGFR at time A in model 1 and at time D in model 2 according to baseline eGFR categories G1, G2, and ≥ G3. The same approach was used to describe the association between IT and the probability of AKI and its interaction with baseline eGFR categories G1, G2, and ≥ G3 in model 3, and to describe the association between IT and the probability of new-onset CKD stage ≥3 at time D in model 4.

Due to missing data (26.6% for AKI, 9.9% for IT, and 54.3 and 47.3% for relative changes in eGFR from baseline at times D and E, respectively), regression models were based on the pooled estimates from 100 imputed datasets. We used the MICE package by R to implement multiple imputations by chained equations [30, 31]. All outcome and independent variables included in the subsequent analyses and additional variables that could provide useful information were used. A *p*-value < 0.05 was considered statistically significant.

## Results

Epidemiological, oncological, and surgical characteristics of the treated patient cohort, subdivided into the LNSS approach group and ONSS approach group, are shown in Table 1. Statistically significant differences between the LNSS and ONSS groups were seen in terms of the preoperative haemoglobin level, tumour diameter, tumour stage, and baseline renal function. Baseline eGFR was higher for the LNSS-RI group compared to the ONSS-RI group (85.5 [IQR, 72.1–96.2] vs. 75.4 [IQR, 61.5–90.2] mL/min; *p* = 0.03), but did not differ between the NSS-RI group and NSS-NRI group (79.5 [IQR, 66.3–93.9] vs. 80.4 [IQR, 59.4–91.1] mL/min; *p* = 0.36). In the NSS-C group, 18.5% of patients had eGFR category ≥G3 at the time of surgery. Hence, the proportion of patients with eGFR category ≥G3 was 13.7% in the LNSS cohort and 22.7% in the ONSS cohort (*p* = 0.01) at baseline. This distribution was similar for subgroups LNSS-RI and ONSS-RI, with 11.9 and 22.8% (*p* = 0.001) having eGFR category ≥G3 at the time of surgery, respectively. There was an insignificantly higher rate of patients with eGFR category ≥G3 in the NSS-NRI group compared to the NSS-RI group (24.6% vs. 16.9%; *p* = 0.17).

**Table 1:** Epidemiological, oncological, and surgical characteristics

	Overall (*n* = 444)	LNSS (*n* = 211)	ONSS (*n* = 233)	*p*-value
a Epidemiological characteristics				
Age years	63 (54–68)	63 (53–68)	63 (55–68)	0.99
Sex m/f (%)	331 / 113 (74.5 / 25.5)	165 / 46 (78.2 / 21.8)	166 / 67 (71.2 / 28.8)	0.09
BMI (kg/m^2^)	26.9 (24.7–29.6)	27.1 (25.1–29.7)	26.8 (24.3–29.6)	0.21
Charlson comorbidity score	2 (0–2)	2 (0–2)	2 (0–2)	0.74
eGFR mL/min/1.73 m^2 a^	80.0 (65.1–93.1)	82.0 (69.9–94.9)	76.9 (61.5–91.1)	0.004
Serum creatinine mg/dL	0.97 (0.83–1.14)	0.93 (0.82–1.08)	1.0 (0.85–1.2)	0.03
eGFR category ^b^ (%)				0.04
G1	134 (30.2)	70 (33.2)	64 (27.5)	0.22
G2	223 (50.2)	111 (52.6)	112 (48.1)	0.41
G3	73 (16.4)	24 (11.4)	49 (21.0)	0.005
G4	9 (2.0)	5 (2.4)	4 (1.7)	0.90
G5	0 (0.0)	0 (0.0)	0 (0.0)	–
No data	5 (1.1)	1 (0.4)	4 (1.7)	0.22
Haemoglobin g/dL	14.5 (13.6–15.4)	14.7 (13.8–15.5)	14.4 (13.5–15.3)	0.02
Blood pressure categories ^c^ (%)				0.92
Optimal blood pressure	36 (8.1)	16 (7.6)	20 (8.6)	
Normal blood pressure	84 (18.9)	30 (14.2)	54 (23.2)	
High-normal blood pressure	75 (16.9)	32 (15.2)	43 (18.5)	
Grade 1 hypertension [mild]	132 (29.7)	60 (28.4)	72 (30.9)	
Grade 2 hypertension [moderate]	50 (11.3)	16 (7.6)	34 (14.6)	
Grade 3 hypertension [severe]	10 (2.3)	5 (2.4)	5 (2.1)	
No data	57 (12.8)	52 (24.6)	5 (2.1)	
b Tumour characteristics				
RCC subtype (%)				0.53
Clear cell	303 (68.2)	141 (66.8)	162 (69.5)	
Papillary	94 (21.2)	52 (24.6)	42 (18.0)	
Chromophobic	34 (7.7)	14 (6.6)	20 (8.6)	
Others ^d^	5 (1.1)	2 (0.9)	3 (1.3)	
No data	8 (1.8)	2 (0.9)	6 (2.6)	
TNM (7th edition UICC 2010) staging (%)				< 0.001
T1a	315 (70.9)	170 (80.6)	145 (62.2)	< 0.001
T1b	83 (18.7)	29 (13.7)	54 (23.2)	0.008
T2a	10 (2.3)	1 (0.5)	9 (3.9)	0.02
T2b	3 (0.7)	0 (0.0)	3 (1.3)	0.10
T3a	22 (5.0)	8 (3.8)	14 (6.0)	0.26
T3b	1 (0.2)	0 (0.0)	1 (0.4)	0.34
T3c	0 (0.0)	0 (0.0)	0 (0.0)	–
T4	1 (0.2)	1 (0.5)	0 (0.0)	0.30
Tx	9 (2.0)	2 (0.9)	7 (3.0)	0.13
Nodal status (%)				0.17
N0	45 (10.1)	17 (8.1)	28 (12.0)	
N1	0 (0.0)	0 (0.0)	0 (0.0)	
Nx	399 (89.1)	194 (91.9)	205 (88.0)	
Metastatic status (%)				
M0	444 (100.0%)	211 (100.0)	233 (100.0)	–
M1	0 (0.0)	0 (0.0)	0 (0.0)	–
Mx	0 (0.0)	0 (0.0)	0 (0.0)	–
Grading (%)				0.20
G1	77 (17.3)	39 (18.5)	38 (16.3)	
G2	300 (67.6)	150 (71.1)	150 (64.4)	
G3	36 (8.1)	13 (6.2)	23 (9.9)	
G4	2 (0.5)	0 (0.0)	2 (0.9)	
No data	29 (6.5)	9 (4.3)	20 (8.6)	
Surgical margin (%)				
R0	396 (89.2)	194 (91.9)	202 (86.7)	0.86
R1	15 (3.4)	7 (3.3)	8 (3.4)	0.14
R2	0 (0.0)	0 (0.0)	0 (0.0)	–
Rx	33 (7.4)	10 (4.7)	23 (9.9)	0.04
R+	16 (3.6)	7 (3.3)	9 (3.9)	0.68
Tumour diameter cm	3.0 (2.2–4.0)	2.7 (2.0–3.5)	3.5 (2.5–4.5)	< 0.001
Tumour diameter category cm (%)				
≤ 4 cm	357 (80.4)	185 (87.7)	172 (73.8)	< 0.001
> 4 - < 7 cm	74 (16.7)	25 (11.8)	49 (21.0)	0.01
≥ 7 – < 10 cm	10 (2.3)	1 (0.5)	9 (3.9)	0.02
≥ 10 cm (%)	3 (0.7)	0 (0.0)	3 (1.3)	0.10
Tumour location				
Side (%)				0.81
Right	233 (52.5)	112 (53.1)	121 (51.9)	
Left	211 (47.5)	99 (46.9)	112 (48.1)	
Bilateral	0 (0.0)	0 (0.0)	0 (0.0)	
Quantity (%)				0.50
Unilocally	430 (96.8)	206 (97.6)	224 (96.1)	
Multilocally	13 (2.9)	5 (2.4)	8 (3.4)	
No data	1 (0.2)	0 (0.0)	1 (0.4)	
Vertical locus (%)				
Located in upper renal section	119 (26.8)	59 (28.0)	60 (25.8)	0.61
Located in middle renal section	168 (37.8)	86 (40.8)	82 (35.2)	0.24
Located in lower renal section	159 (35.8)	65 (30.8)	94 (40.3)	0.03
No data	5 (1.1)	2 (0.9)	3 (1.3)	0.74
Horizontal locus (%)				< 0.001
Centrally located	122 (27.5)	41 (19.4)	81 (34.8)	< 0.001
Peripherically located	313 (70.5)	164 (77.7)	149 (63.9)	< 0.001
No data	9 (2.0)	6 (2.8)	3 (1.3)	0.25
c Surgical characteristics				
Operative time min	185.0 (145.0–230.0)	205.0 (160.0–245.0)	160.0 (130.0–207.0)	< 0.001
Vessel clamping (%)				< 0.001
Ischaemia	343 (77.3)	185 (87.7)	158 (67.8)	< 0.001
No Ischaemia	57 (12.8)	9 (4.3)	48 (20.6)	< 0.001
No data	44 (9.9)	17 (8.1)	27 (11.6)	0.21
Ischaemia time min	24 (18–32)	25 (19–34)	23 (17–30)	0.04
Ischaemia time < 15 min / ≥ 15 min (%)	41 / 302 (9.2 / 68.0)	19 / 166 (9.0 / 78.7)	22 / 136 (9.4 / 58.4)	0.30
Ischaemia time < 20 min / ≥ 20 min (%)	99 / 244 (22.3 / 55.0)	51 / 134 (24.2 / 63.5)	48 / 110 (20.6 / 47.2)	0.57
Ischaemia time < 25 min / ≥ 25 min (%)	174 /169 (39.2 /38.1)	84 / 101 (39.8 / 47.9)	90 / 68 (38.6 / 29.2)	0.03
Ischaemia time < 30 min / ≥ 30 min (%)	231 /112 (52.0 /25.2)	118 / 67 (55.9 / 31.8)	113 / 45 (48.5 / 19.3)	0.13
Ischaemia time < 35 min / ≥ 35 min (%)	269 / 74 (60.6 /16.7)	141 / 44 (66.8 / 20.9)	128 / 30 (54.9 /12.9)	0.28
Lymphadenctomy (%)	45 (10.1)	17 (8.1)	28 (12.0)	0.17
Adrenalectomy (%)	7 (1.6)	3 (1.4)	4 (1.7)	0.92
Intraoperative transfusion rate (%)	25 (5.6)	4 (1.9)	21 (9.0)	< 0.001
Transfusion bags	2 (1–2)	1 (1–1.75)	2 (1–2)	0.12
Estimated intraoperative blood loss mL	200 (100–500) *n* = 88	150 (50–425) *n* = 49	400 (200–800) *n* = 39	< 0.001
Surgical approach (%)				< 0.001
Retroperitoneal	162 (36.5)	0 (0.0)	162 (69.5)	< 0.001
Transperitoneal	275 (61.9)	211 (100.0)	64 (27.5)	< 0.001
No data	7 (1.6)	0 (0.0)	7 (3.0)	0.01
Preoperative prophylactic ureteric stent implantation (%)	72 (16.2)	54 (25.6)	18 (7.7)	< 0.001
Postoperative ureteric stent implantation (%)	14 (3.2)	5 (2.4)	9 (3.9)	0.37
Intraoperative complications (%)	64 (14.4)	34 (16.1)	30 (12.9)	0.33
Postoperative complications ^e^ (%)	140 (31.5)	49 (23.2)	91 (39.1)	< 0.001
Clavien-Dindo score ≥ 3 (%)	40 (9.0)	16 (7.6)	24 (10.3)	0.32
Hospitalization d	7 (5–9)	6 (5–8)	8 (6–10)	< 0.001

(a) Patient characteristics, epidemiological data, clinical and renal parameters, (b) tumour characteristics, and (c) surgical characteristics of the overall study population (NSS-C) and in the LNSS and ONSS subgroups

Continuous data are shown as median with interquartile range (IQR)

*LNSS* laparoscopic nephron-sparing surgery, *ONSS* open nephron-sparing surgery, *n* number, *m* male, *f* female, *RCC* renal cell cancer, *BMI* body mass index, *eGFR* estimated glomerular filtration rate, *CT* computed tomography, *MRI* magnetic resonance imaging, *UICC* Union Internationale Contre le Cancer, *d* days

^a^Calculated according to the MDRD (Modification of Diet in Renal Disease) formula

^b^According to the Kidney Disease: Improving Global Outcomes (KDIGO) criteria

^c^According to the 1999 World Health Organization International Society of Hypertension. Guidelines for the management of hypertension

^d^Chromophile, unclassifiable, or dedifferentiated carcinoma

^e^According to the Clavien-Dindo classification

The median tumour diameter was 0.8 cm smaller for the LNSS group compared to the ONSS group (*p* <  0.001) and was 0.5 cm smaller for the NSS-NRI group compared to the NSS-RI group (2.7 [IQR 2.0–3.5] vs. 3.2 [IQR 2.2–4.0] cm; *p* = 0.04). Operative time was 45 min longer (*p* <  0.001) and IT was 2 min shorter (*p* = 0.04) for the LNSS group. The ischaemia rate was 87.8% for the LNSS group and 67.8% for the ONSS group (*p* <  0.001). In total, 57 patients (12.8%) underwent ZI surgery; 38.5% of the patients in the NSS-C group developed AKI postoperatively as follows: stage 1, 33.9%; stage 2, 4.3%; and stage 3, 0.3%. Significant differences were observed for the surgical approach, baseline eGFR categories, and IT (Table 2a). AKI stages 1, 2, and 3 were detected in 40.2, 7.5, and 0.9% of patients in the ONSS cohort and in 28.3, 2.0, and 0.0% of patients in the LNSS cohort, respectively (*p* = 0.001).

**Table 2:** Rate (%) of AKI and new-onset CKD stage ≥3

a	Rate of AKI (%)	*p*-value
NSS-C	38.5	
NSS-RI	37.8	0.45
NSS-NRI	31.7	
LNSS-RI	30.3	0.003
ONSS-RI	48.6	
NSS-G1	24.0	< 0.001
NSS-G2	41.9	
NSS ≥ G3	56.9	
IT ≥20 min	43.4	0.005
IT < 20 min	24.7	
b	Rate of new-onset CKD stage ≥3 (%)	*p*-value
NSS-C	27.9	
NSS-RI	26.3	0.63
NSS-NRI	30.8	
LNSS-RI	20.9	0.08
ONSS-RI	33.3	
NSS-AKI	35.2	0.02
NSS-NAKI	17.7	
NSS-G1	7.9	< 0.001
NSS-G2	37.3	
IT ≥30 min	38.0	0.02
IT < 30 min	20.9	

Rate (%) of (a) AKI within 48 h postoperatively and of (b) new-onset CKD stage ≥ 3 within the latest recorded follow-up of a median of 50 (IQR, 35–81) months p.o. in the overall NSS cohort (NSS-C) and according to different subgroups

Subgroups: NSS group with intraoperative renal ischaemia (NSS-RI), NSS group without intraoperative renal ischaemia (NSS-NRI), LNSS group with intraoperative renal ischaemia (LNSS-RI), ONSS group with intraoperative renal ischaemia (ONSS-RI), NSS group with development of postoperative AKI (NSS-AKI), NSS group without development of postoperative AKI (NSS-NAKI), NSS group with baseline eGFR category G1 (NSS-G1), NSS group with baseline eGFR category G2 (NSS-G2), and NSS group with baseline eGFR category ≥ G3 (NSS ≥ G3), and NSS groups with different ischaemia times (IT)

*AKI* acute kidney injury, *CKD* chronic kidney disease, *p.o.* postoperatively, *NSS* nephron-sparing surgery, *LNSS* laparoscopic nephron-sparing surgery, *ONSS* open nephron-sparing surgery

**Fig. 1:**
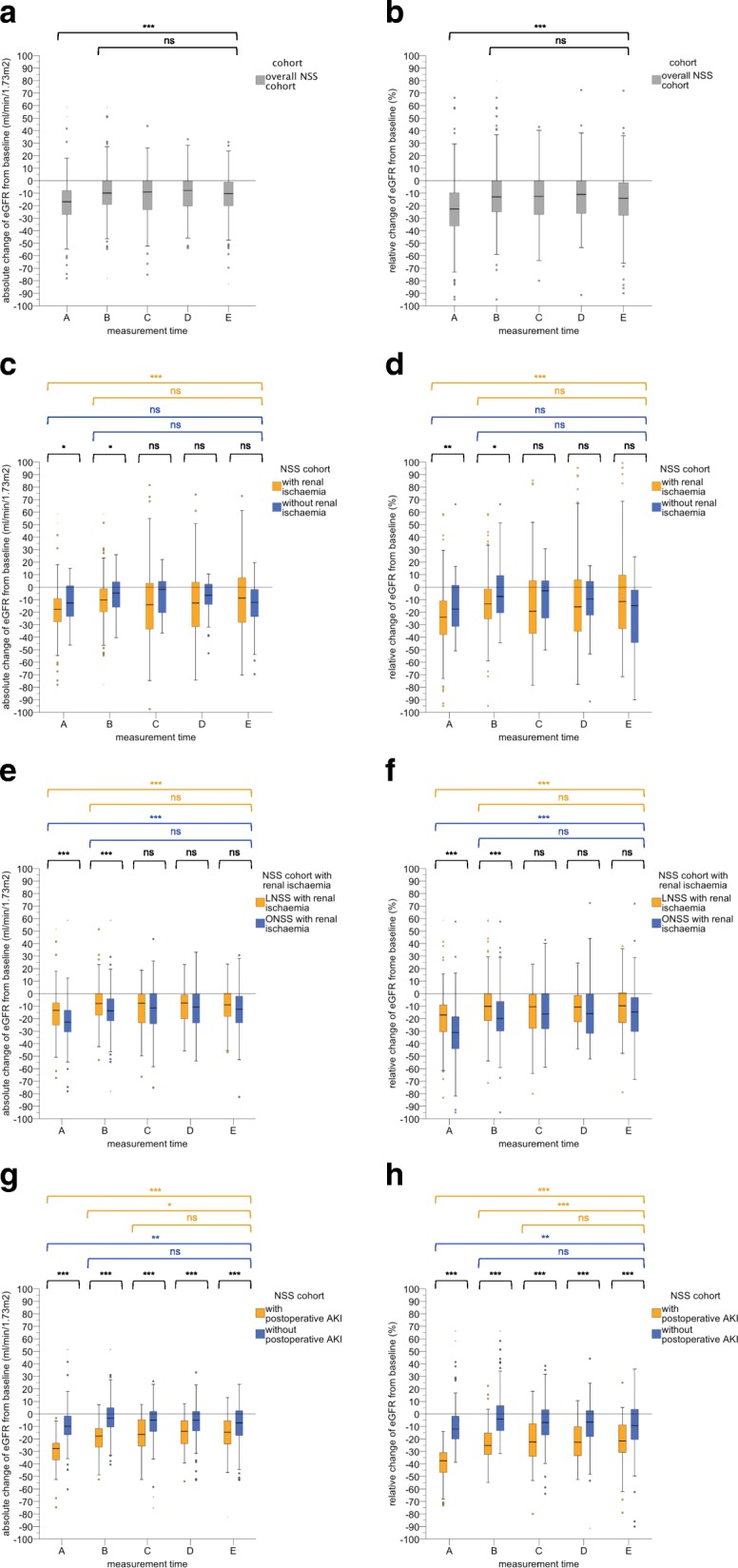
Box plots showing the postoperative course of the absolute (**a**/**c**/**e**/**g**/**i**) and relative (**b**/**d**/**f**/**h**/**j**) change (%) in eGFR at measurement times A-E for (**a**/**b**) the overall NSS cohort (NSS-C), (**c**/**d**) the NSS group with intraoperative renal ischaemia (NSS-RI) and without intraoperative renal ischaemia (NSS-NRI), (**e**/**f**) the LNSS group with intraoperative renal ischaemia (LNSS-RI), the ONSS group with intraoperative renal ischaemia (ONSS-RI), (**g**/**h**) the NSS group with postoperative AKI (NSS-AKI), the NSS group without postoperative AKI (NSS-NAKI), and (**i**/**j**) NSS group with a baseline eGFR category G1 (NSS-G1), NSS group with a baseline eGFR category G2 (NSS-G2), and NSS group with a baseline eGFR category ≥G3 (NSS ≥ G3). Definition of measurement times **a**-**e**: (**a**) highest change in eGFR from baseline during the planned hospital stay at a median of 1 day postoperatively (IQR, 1–2), (**b**) change in eGFR from baseline prior to discharge from hospital at a median of 4 days postoperatively (IQR, 2–6), (**c**) change in eGFR from baseline at a median of 47 days postoperatively (IQR, 30–105), (**d**) a median of 13 months postoperatively (IQR, 12–15), and (**e**) a median of 50 months postoperatively (IQR, 35–81). Asterisks indicate significant changes from baseline in the level of absolute and relative changes in eGFR over the course of the observation period (Friedman’s test as a post hoc pairwise multiple comparison test) or between the compared groups at each measurement time (non-parametric Mann-Whitney U test). * *p* <  0.05, ** *p* <  0.01, *** *p* <  0.001, (ns) not significant. eGFR, estimated glomerular filtration rate; NSS, nephron-sparing surgery; LNSS, laparoscopic nephron-sparing surgery; ONSS, open nephron-sparing surgery; AKI, acute kidney injury; IQR, interquartile range

Table 3 and Fig. 1a-j show the absolute and relative (%) changes in eGFR from baseline at postoperative measurement times A-E in the NSS-C group (Fig. 1a/b) and subgroups according to the application of ischaemia (Fig. 1c/d), surgical approach (Fig. 1e/f), and occurrence of postoperative AKI (Fig. 1g/h), and according to baseline renal function (Fig. 1i/j). The highest relative renal function reduction was seen at a median of 1 day postoperatively (IQR, 1–2) (time A) in the NSS-C group and in all subgroups; all compared subgroups showed significantly different eGFR reductions at time A. No statistically significant difference between the compared subgroups was seen at a median of 13 months postoperatively (IQR, 12–15) (time D) and at the last follow-up, which occurred at a median of 50 months postoperatively (IQR, 35–81) (time E). In contrast, the NSS-AKI group consistently showed significantly higher absolute and relative reductions of eGFR compared to the NSS-NAKI group at all postoperative measurement times (*p* <  0.001).

**Table 3:** Absolute and relative (%) changes in eGFR

Cohort/ subgroup	Measurement time A (1 [1–2] day p.o.)	Measurement time B (4 [2–6] days p.o.)	Measurement time C (47 [30–105] days p.o.)	Measurement time D (13 [12–15] months p.o.)	Measurement time E (50 [35–81] months p.o.)	*p*-value
NSS-C	−16.8 [−27.0 - −7.9]	−9.9 [−19.1–0.0]	−9.1 [−23.2–0.0]	−7.8 [−20.4–0.25]	−10.4 [−20.2 - −1.2]	< 0.001
*n* = 444 (100.0%)	−22.6% [−36.2% - −9.7%]	−13.0% [−24.9%–0.0%]	−12.5% [−27.2%–0.0%]	−11.0% [−26.3%–0.3%]	−14.1% [−27.7% - −1.3%]	< 0.001^*^
	*n* = 436 (98.2%)	*n* = 436 (98.2%)	*n* = 184 (41.4%)	*n* = 203 (45.7%)	*n* = 234 (52.7%)	
NSS-RI	− 17.7 [− 27.8 - −9.1]	−10.2 [− 19.8 - −1.2]	−9.7 [− 23.8–0.1]	−8.6 [−21.0–0.6]	−10.0 [− 19.6–0.67]	< 0.001
*n* = 343 (100.0%)	− 24.0% [− 38.0% - −10.9%]	− 13.7% [− 25.5%- −1.5%]	− 19.3% [− 37.2%–5.5%]	− 15.8% [− 35.3%–6.1%]	− 11.5% [− 33.4%–9.8%]	< 0.001^*^
	*n* = 337 (98.3%)	*n* = 337 (98.3%)	*n* = 148 (43.1%)	*n* = 161 (46.9%)	n = 183 (53.4%)	
NSS-NRI	−12.6 [− 23.8–1.3]	−4.7 [− 17.5–4.7]	−1.9 [− 21.9–5.7]	−6.6 [− 14.0–2.6]	− 12.1 [− 25.7 - −1.2]	0.15
*n* = 57 (100.0%)	− 17.5% [−31.9%–2.1%]	− 7.4% [− 21.3%–9.5%]	−2.9% [− 25.0%–6.6%]	− 9.4% [− 22.7%–5.3%]	− 14.8% [− 44.5% - −1.4%]	0.15^*^
	*n* = 57 (100.0%)	*n* = 57 (100.0%)	*n* = 25 (43.9%)	*n* = 28 (49.1%)	*n* = 34 (59.6%)	
*p*-value	0.01	0.03	0.1	0.43	0.28	
	0.007^*^	0.01^*^	0.07^*^	0.35^*^	0.13^*^	
LNSS-RI	−13.3 [− 25.3 - −7.1]	−8.0 [− 17.5–0.0]	−7.7 [− 23.4 - −0.4]	−7.4 [− 20.1 - −1.0]	−9.0 [− 18.4 - −0.1]	< 0.001
*n* = 185 (100.0%)	− 17.0% [−30.5% - −9.0%]	− 10.1% [− 21.6%–0.0%]	− 10.5% [− 27.7% - −0.5%]	− 10.8% [− 23.0% - −1.3%]	− 9.8% [− 23.4%–1.8%]	< 0.001^*^
	*n* = 183 (98.9%)	*n* = 183 (98.9%)	*n* = 76 (41.0%)	*n* = 90 (48.6%)	*n* = 102 (55.1%)	
ONSS-RI	−22.8 [− 31.0 - −13.2]	− 13.7 [− 22.1 - −4.1]	−11.5 [− 24.2–0.0]	−10.7 [− 23.4 - −0.3]	− 12.6 [− 23.7 - −2.0]	< 0.001
*n* = 158 (100.0%)	− 31.1% [− 44.1% - −18.5%]	− 19.8% [−29.9% - −6.1%]	− 16.2% [−28.0% - −16.2%]	− 15.9% [−32.5% - −0.3%]	− 14.7% [− 30.3% - −2.9%]	< 0.001^*^
	*n* = 154 (97.5%)	*n* = 154 (97.5%)	*n* = 72 (45.6%)	*n* = 71 (44.9%)	*n* = 81 (51.6%)	
*p*-value	< 0.001	< 0.001	0.58	0.49	0.06	
	< 0.001^*^	< 0.001^*^	0.45^*^	0.37^*^	0.06^*^	
NSS-AKI	−27.7 [−36.8 - −23.3]	− 17.3 [− 26.6 - −11.7]	−16.4 [− 25.9 - − 4.5]	−13.8 [− 24.0 - −5.2]	−14.7 [− 24.5 - −5.4]	< 0.001
*n* = 126 (100.0%)	−37.5% [− 46.8% - −30.9%]	− 25.2% [− 32.4% - −15.2%]	− 22.4% [−34.0% - −7.7%]	− 22.7% [− 33.5% - −10.2%]	− 21.6% [− 31.0% - −8.8%]	< 0.001^*^
	*n* = 126 (100.0%)	*n* = 126 (100.0%)	*n* = 60 (47.6%)	*n* = 65 (51.5%)	*n* = 68 (53.9%)	
NSS-NAKI	−9.8 [− 16.8- −1.6]	−3.4 [− 10.4–4.9]	−5.0 [− 14.0–2.0]	−5.0 [− 13.5–2.0]	−7.2 [− 17.3–3.0]	< 0.001
*n* = 201 (100.0%)	− 12.1% [− 20.1% - −1.8%]	− 4.2% [− 13.3% - −6.8%]	− 6.8% [− 17.1%–3.4%]	− 6.6% [− 18.3%–2.6%]	− 9.4% [− 21.2%–4.4%]	0.004^*^
	*n* = 201 (100.0%)	*n* = 201 (100.0%)	*n* = 80 (39.8%)	*n* = 92 (45.8%)	*n* = 109 (54.2%)	
*p*-value	< 0.001	< 0.001	< 0.001	< 0.001	< 0.001	
	< 0.001^*^	< 0.001^*^	< 0.001^*^	< 0.001^*^	< 0.001^*^	
NSS-G1	− 18.0 [− 32.7 - −7.5]	− 10.9 [− 24.4 - −0.6]	−20.4 [− 30.3 - − 5.4]	−10.3 [− 31.0 - − 0.5]	−13.0 [− 31.9 - − 4.9]	0.04
*n* = 134 (100.0%)	− 19.5% [− 30.6% - −7.4%]	− 10.6% [− 22.9% - −0.7%]	− 21.0% [− 28.7% - −5.7%]	− 10.6% [− 29.8% - −0.1%]	− 13.2% [− 29.8% - −5.1%]	0.04^*^
	*n* = 133 (99.0%)	*n* = 133 (99.0%)	*n* = 42 (31.3%)	*n* = 46 (34.3%)	*n* = 62 (46.3%)	
NSS-G2	−17.8 [− 27.6 - −8.6]	−10.4 [− 18.0 - −1.1]	−9.7 [− 22.7 - − 0.1]	−10.0 [− 21.5 - − 0.3]	−10.2 [− 19.6 - − 0.7]	< 0.001
*n* = 223 (100.0%)	− 24.5% [− 36.5% - −11.0%]	− 13.4% [− 24.5% - −1.2%]	− 12.3% [− 27.2% - −0.1%]	− 12.5% [− 27.2% - −0.4%]	− 14.1% [− 24.0% - −0.9%]	< 0.001^*^
	*n* = 221 (99.0%)	*n* = 221 (99.0%)	*n* = 104 (46.6%)	*n* = 120 (53.8%)	*n* = 131 (58.7%)	
NSS ≥ G3	−13.6 [− 22.8 - −3.4]	−5.1 [− 13.9–1.3]	−3.4 [− 9.1–2.4]	− 4.8 [− 10.6–2.1]	−7.2 [− 15.3–0.9]	< 0.001
*n* = 82 (100.0%)	− 29.3% [− 46.4% - −9.3%]	− 13.5% [− 29.3%–4.1%]	− 7.9% [− 17.1% - 7.4%]	− 9.0% [− 22.0%–4.6%]	− 14.7% [− 29.9%–1.8%]	< 0.001^*^
	*n* = 82 (100.0%)	*n* = 82 (100.0%)	*n* = 38 (46.3%)	*n* = 37 (45.1%)	*n* = 42 (51.2%)	
*p*-value	0.02	0.007	< 0.001	0.03	0.02	
	0.004^*^	0.78^*^	0.03^*^	0.43^*^	0.6^*^	

Absolute and relative (%) changes in eGFR from baseline for the overall NSS cohort (NSS-C) and for different subgroups at different postoperative median measurement times (A-E)

Subgroups: NSS cohort with intraoperative renal ischaemia (NSS-RI), NSS cohort without intraoperative renal ischaemia (NSS-NRI), LNSS with intraoperative renal ischaemia (LNSS-RI), ONSS cohort with intraoperative renal ischaemia (ONSS-RI), NSS cohort with development of postoperative AKI (NSS-AKI), NSS cohort without development of postoperative AKI (NSS-NAKI), NSS cohort with baseline eGFR category G1 (NSS-G1), NSS cohort with baseline eGFR category G2 (NSS-G2), and NSS cohort with baseline eGFR category ≥ G3 (NSS ≥ G3)

Estimated glomerular filtration rate (eGFR) values were calculated according to the MDRD (Modification of Diet in Renal Disease) formula. Data are shown as median and interquartile range [IQR]

*NSS* nephron-sparing surgery, *LNSS* laparoscopic nephron-sparing surgery, *ONSS* open nephron-sparing surgery, *n* numbers investigated, *p.o.* postoperative

^*^P-values refer to relative (%) values

Regarding the complete follow-up period, the rate of new-onset CKD stage ≥3 was + 27.9% for the NSS-C group. Table 2b summarises data for different subgroups.

Results of the multiple linear and logistic regression models investigating predictors for the postoperative short-term (model 1) and long-term (model 2) relative changes in renal function or postoperative development of AKI (model 3) and new-onset CKD stage ≥3 (model 4) are summarised in Table 3a-d.

In regression model 1 (Table 4a), baseline eGFR, ONSS, BMI, IT, major complications, and operative time were independent predictors of a greater short-term relative decrease in eGFR from baseline at time A (1 day postoperatively; IQR, 1–2 days). In regression model 2 (Table 4b), baseline eGFR, tumour diameter, and higher relative decrease of the eGFR at time A (outcome variable in model 1) were all significantly related to greater long-term relative decreases in eGFR at time D (13 months postoperatively; IQR, 12–15 months).

**Table 4:** Multiple linear regression analysis

a (model 1)	Regression coefficient β	Multiple linear regression 95% CI	*p*-value
Baseline eGFR (mL/ml/1.73 m^22^)	- 0.20	- 0.38 - − 0.02	0.03
Baseline Haemoglobin (mg/dL)	0.51	- 0.86 - 1.89	0.46
Tumour diameter (cm)	0.67	- 0.43 - 1.76	0.24
Tumour locus central (ref.) vs. peripheral	0.43	- 3.80 - 4.67	0.84
Surgical approach LNSS (ref.) vs. ONSS	- 13.48	- 17.65 - − 9.32	< 0.001
Sex male (ref.) vs. female	- 3.28	- 7.80 - 1.25	0.16
Age (years)	- 0.17	- 0.36 - 0.01	0.06
BMI (kg/m^2^)	- 0.88	- 1.36 - − 0.41	< 0.001
Hypertension no (ref.) vs. yes	- 0.78	- 4.75 - 3.18	0.70
Ischaemia time (min)	- 0.27	- 0.41 - − 0.13	< 0.001
Operative time (min)	- 0.06	- 0.09 - − 0.03	< 0.001
Preoperative ureter stenting no (ref.) vs. yes	- 0.46	- 5.64 - 4.71	0.86
Intraoperative blood transfusions no (ref.) vs. yes	- 3.29	- 11.91 - 5.33	0.45
Postoperative complications no (ref.) vs. yes	- 3.36	- 8.42 - 1.70	0.19
Clavien-Dindo score < 3 (ref.) vs. ≥ 3	- 10.98	- 18.47 - − 3.48	0.004
b (model 2)	Regression coefficient β	Multiple linear regression 95% CI	*p*-value
Baseline eGFR (mL/ml/1.73 m^22^)	- 0.29	- 0.49 - − 0.09	0.005
Baseline Haemoglobin (mg/dL)	- 0.32	- 1.95 - 1.31	0.70
Relative change of eGFR from baseline at time A (%)	0.18	0.03 - 0.33	0.02
AKI 48 h p.o. no (ref.) vs. yes	- 2.11	- 9.01 - 4.79	0.55
Tumour diameter (cm)	- 1.76	- 2.87 - − 0.66	0.002
Tumour locus central (ref.) vs. peripheral	- 0.30	- 5.14 - 4.54	0.90
Surgical approach LNSS (ref.) vs. ONSS	1.13	- 4.17 - − 6.44	0.67
Sex male (ref.) vs. female	1.63	- 3.43 - 6.70	0.53
Age (years)	- 0.10	- 0.33 - 0.13	0.40
BMI (kg/m2)	0.15	- 0.39 - 0.70	0.58
Hypertension no (ref.) vs. yes	- 2.11	- 6.82 - 2.60	0.38
Ischaemia time (min)	0.03	- 0.14 - 0.21	0.72
Operative time (min)	0.01	- 0.03 - 0.05	0.54
Preoperative ureter stenting no (ref.) vs. yes	- 4.35	- 10.46 - 1.77	0.16
Intraoperative blood transfusions no (ref.) vs. yes	4.12	- 6.29 - 14.52	0.44
Postoperative complications no (ref.) vs. yes	1.20	- 4.77 - 7.18	0.69
Clavien-Dindo score < 3 (ref.) vs. ≥ 3	4.43	- 4.28 - 13.14	0.32
c (model 3)	OR	Multiple logistic regression 95% CI	*p*-value
Baseline eGFR (mL/ml/1.73 m^22^)	0.99	0.96 - 1.01	0.30
Baseline Haemoglobin (mg/dl)	0.85	0.70 - 1.03	0.10
Tumour diameter (cm)	0.94	0.81 - 1.08	0.35
Tumour locus central (ref.) vs. peripheral	1.20	0.70 - 2.05	0.51
Surgical approach LNSS (ref.) vs. ONSS	3.87	2.17 - 6.92	< 0.001
Sex male (ref.) vs. female	2.51	1.35 - 4.67	0.004
Age (years)	1.01	0.99 - 1.04	0.26
BMI (kg/m^2^)	1.13	1.06 - 1.21	< 0.001
Hypertension no (ref.) vs. yes	1.05	0.63 - 1.74	0.85
Ischaemia time (min)	1.02	1.00 - 1.04	0.046
Operative time (min)	1.01	1.00 - 1.01	0.002
Preoperative ureter stenting no (ref.) vs. yes	0.92	0.46 - 1.83	0.81
Intraoperative blood transfusions no (ref.) vs. yes	0.73	0.22 - 2.45	0.61
Postoperative complications no (ref.) vs. yes	1.79	0.92 - 3.48	0.08
Clavien-Dindo score < 3 (ref.) vs. ≥ 3	2.14	0.68 - 6.72	0.19
d (model 4)	OR	Multiple logistic regression 95% CI	*p*-value
Baseline eGFR (mL/ml/1.73 m^2^)	0.89	0.85 - 0.92	< 0.001
Baseline Haemoglobin (mg/dL)	0.99	0.73 - 1.35	0.95
Relative change of eGFR from baseline at time A (%)	0.98	0.98 - 1.01	0.12
AKI 48 h p.o. no (ref.) vs. yes	1.23	0.39 - 3.85	0.72
Tumour diameter (cm)	0.93	0.71 - 1.21	0.58
Tumour locus central (ref.) vs. peripheral	1.35	0.56 - 3.15	0.49
Surgical approach LNSS (ref.) vs. ONSS	1.69	0.67 - 4.24	0.26
Sex male (ref.) vs. female	0.63	0.24 - 1.67	0.35
Age (years)	0.99	0.95 - 1.04	0.75
BMI (kg/m^2^)	0.97	0.87 - 1.07	0.50
Hypertension no (ref.) vs. yes	1.62	0.66 - 4.00	0.29
Ischaemia time (min)	1.01	0.98 - 1.04	0.55
Operative time (min)	1.00	0.99 - 1.01	0.86
Preoperative ureter stenting no (ref.) vs. yes	1.26	0.41 - 3.86	0.68
Intraoperative blood transfusions no (ref.) vs. yes	0.95	0.08 - 11.05	0.97
Postoperative complications no (ref.) vs. yes	0.67	0.22 - 2.00	0.47
Clavien-Dindo score < 3 (ref.) vs. ≥ 3	1.37	0.22 - 8.41	0.73

Multiple linear regression analysis for models 1 and 2 including ischaemia time as a continuous variable investigating predictors of the relative change (%) of eGFR from baseline at (a) measurement time A (median, 1 day p.o.; IQR, 1–2) and at (b) at measurement time D (median, 13 months p.o.; IQR 12–15), and multiple logistic regression analysis for models 3 and 4 including ischaemia time as a continuous variable investigating (c) predictors for the development of postoperative AKI within 48 h p.o. and (d) predictors for the development of postoperative new-onset CKD stage ≥ 3 (eGFR < 60 mL/min/1.73 m2) within measurement time D

The regression models are based on pooled estimates from 100 imputed datasets. A *p*-value < 0.05 is regarded as statistically significant

*eGFR* estimated glomerular filtration rate, *CKD* chronic kidney disease, *AKI* acute kidney injury, *LNSS* laparoscopic nephron-sparing surgery, *ONSS* open nephron-sparing surgery, *BMI* body mass index, *OR* odds ratio

Risk factors for postoperative AKI within 48 h in model 3 (Table 4c) were ONSS, male sex, higher BMI, longer IT, and longer operative time.

Including only patients with preoperative eGFR category <G3, regression model 4 (Table 4d) showed that lower baseline eGFR was a significant risk factor for development of new-onset CKD stage ≥3 at time D.

In our regression analyses using IT as an independent categorical variable, renal ischaemia compared to ZI showed a significantly greater relative decrease in short-term renal function in model 1 (regression coefficient [ß], − 9.4; 95% CI, − 14.6 to − 4.2; *p* <  0.001); however, no differences were seen in models 2–4. In addition, depending on the baseline eGFR categories, ZI did not affect the development of postoperative AKI in a significantly different manner (data not shown).

**Fig. 2:**
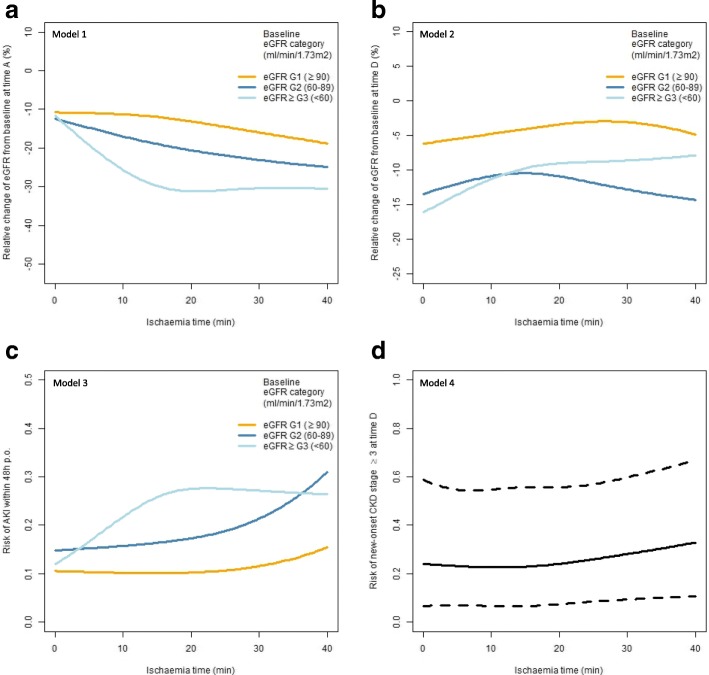
Plots of natural cubic splines including zero ischaemia to estimate the correlation between ischaemia time and (**a**) the relative (%) change in eGFR from baseline at time A (highest change in eGFR during the planned hospital stay prior to discharge (median, 1 day p.o.; IQR, 1–2) in model 1, (**b**) at time D (median, 13 months p.o.; IQR, 12–15) in model 2, (**c**) correlation between ischaemia time and the risk of AKI within 48 h p.o. in model 3 and its interaction with baseline renal function, respectively, and (**d**) correlation between ischaemia time and the risk of new-onset CKD stage ≥3 at time D. CKD, chronic kidney disease; eGFR, estimated glomerular filtration rate; p.o., postoperatively; IQR, interquartile range

Therefore, we used the plot of natural cubic splines (Fig. 2) to estimate the correlation between IT and relative change in eGFR in models 1 and 2 (Fig. 2a and c) and to better demonstrate its interaction with baseline eGFR categories. Additionally, Fig. 2b and d similarly show the correlation between IT and the probability of postoperative AKI and CKD stage ≥3 in models 3 and 4. Based on the results of the regression analysis, some clear assertions can be made regarding the natural cubic splines, including that IT impacts the short-term relative change in eGFR and has greater effects on patients with baseline eGFR categories ≥G3, with the largest effect in this group occurring during the first 15 min of IT (Fig. 2a). The risk for development of AKI within 48 h postoperatively increases with increasing IT, but the effect of IT on AKI risk strongly depends on baseline renal function. Although eGFR category ≥G3 showed a steep increase in the risk for AKI during the first 20 min of ischaemia, the risk for AKI with G1 and, in particular, G2 visibly starts to increase only after 20 min of IT.

Regarding long-term outcomes of NSS, renal function was not significantly affected by IT in any of the baseline eGFR categories; however, reduction in renal function appears to depend on baseline eGFR, with category G1 showing the smallest reduction (Fig. 2c). No strong correlation was seen between the rate of new-onset CKD stage ≥3 and IT (Fig. 2d).

## Discussion

NSS offers similar oncological efficacy for localised RCC as radical nephrectomy and is the current gold standard for the treatment of small renal tumours, even in the presence of a normal contralateral kidney [1, 2]. Recently, the EAU guidelines have been expanded with regard to the recommendation for NSS to include cT2 tumours in selected cases [2]. Approximately 25% of patients with a kidney tumour, normal serum creatinine, and a normal contralateral kidney experience preoperative CKD stage ≥3 [22, 32]. These patients can benefit from the enhanced preservation of renal function after NSS relative to radical nephrectomy, thereby minimising the risk of CKD progression and improving overall survival rates [10, 12, 16, 20, 22, 23, 33]. However, patients treated with NSS have a 16–40% chance of postoperatively developing new-onset CKD stage ≥3 [25, 26]. Despite partial nephrectomy, approximately 90% of the global renal function in patients with bilateral kidneys, and 80% of the global renal function in patients with a solitary kidney are preserved [34]. Key features associated with long-term preservation of renal function include ischaemia type, the amount of healthy renal parenchyma preservation (quantity of nephrons saved), and baseline kidney function (quality of nephrons prior to surgery) [34–40]. Other debated factors related to postoperative renal function are the surgical approach, namely LNSS or ONSS [5, 25, 41, 42], ZI NSS, and the effect of IT [37, 43]. Our study aimed to identify risk factors for the development of AKI and CKD stage ≥3 following NSS. We compared the effects of ONSS and LNSS and the effects of IT and ZI on postoperative short- and long-term renal function in the treatment of RCC patients.

In our cohort of 211 LNSS and 233 ONSS patients (444 patients) with localised RCC, medians of 11.0% and 14.1% reductions in eGFR were detected at medians of 13 months postoperatively (IQR, 12–15) and 50 months postoperatively (IQR, 35–81), respectively. No statistically significant change in eGFR was seen from a median of 47 days postoperatively (IQR, 30–105) until the last follow-up (median, 50 months postoperatively; IQR, 35–81 months) in the overall cohort and in all investigated subgroups. This is in line with previously published data showing little increase in renal function from approximately 6 weeks after NSS [34, 42, 44, 45]. We could not substantiate a statistically significant difference in the relative reduction of renal function during long-term follow-up after NSS according to the surgical approach, application of intraoperative renal ischaemia or ZI, and IT. However, the NSS-NAKI group showed the lowest and the NSS-AKI group showed the highest renal function decrease during long-term follow-up. In our multiple regression model, AKI was not a significant predictor for the relative long-term change in renal function. The main factors of the regression model that predicted renal function at a median of 13 months postoperatively (IQR, 12–15) were baseline eGFR, short-term relative change in renal function at a median of 1 day postoperatively (IQR, 1–2), and tumour diameter. Furthermore, the LNSS group, ZI group, and patients with better baseline kidney function had significantly lower relative reductions in renal function immediately postoperatively. This was consistently reflected in our regression model showing that ONSS, lower baseline eGFR, longer IT, higher BMI, longer operative time, and severe postoperative complications were risk factors for a relative decrease in renal function immediately postoperatively.

Effects of the surgical approach on postoperative renal function are inconsistently described in the literature. Adamy et al. also used the MDRD formula to estimate GFR and showed a more pronounced initial postoperative decrease in renal function with ONSS and slightly but significantly better recovery of the renal function with LNSS [42]. Funahashi et al. also detected a greater decrease in renal function after ONSS [46]. Just recently, Antonelli et al. described a bigger risk for a greater than 25% immediate impaired renal function after ONSS and LNSS when compared to robot-assisted laparoscopic partial nephrectomy with ONSS, showing a bigger odds ratio than LNSS (5.26, *p* <  0.001 vs. 2.86, *p* = 0.004) [24]. However, other human and animal studies were unable to find differences in postoperative renal function after various surgical approaches [6, 39, 47–49]. In contrast, Marszalek et al. showed an 11-fold higher decrease in eGFR 24 h after LNSS compared to ONSS with renal clamping; a similar renal function decrease was found after 3.6 years (− 10.6% vs. -10.9%; *p* = 0.7). However, tumour diameters were equal with LNSS and ONSS, and IT was longer with ONSS in their study. The authors explained their results with evidence of direct and indirect (e.g., renal blood flow) negative impacts of the capnoperitoneum [5, 50]. In contrast, animal experiments have provided indirect evidence suggesting that transient ischaemic preconditioning caused by pneumoperitoneum could limit renal ischaemia/reperfusion injury in laparoscopic surgery [51, 52], potentially indicating a more protective effect with LNSS. However, there was a smaller short-term decrease in renal function in LNSS compared to ONSS in the ZI group (− 9.0 [IQR -23.1 to 4.9] vs. -20.5 [IQR -34.5 to 2.4] %); this difference was not significant (*p* = 0.21).

In our study, the AKI rate was 38.5%, which is similar to previously published data [53]; However, AKI rates after NSS vary between 18 and 54% depending on AKI criteria applied and conditions with solitary or bilateral kidneys [12, 39, 48, 49, 54]. ONSS and male sex were the main risk factors for AKI according to our multiple regression model. There is evidence that longer IT and operative time are risk factors for postoperative AKI after NSS [39, 48, 49, 53], which was confirmed by our data. In our study, every minute of surgery increased the risk of AKI significantly by 1.0%, and every minute of ischaemia increased the risk by 2.0%. In our multiple regression analysis, ZI was significantly associated with a reduced short-term decrease of renal function, but this did not result in a significantly lower risk of AKI (*p* = 0.4).

Additionally, Rajan et al. previously showed that lower baseline eGFR increases the risk for AKI after NSS [53]. In our study, AKI rates were 1.7-fold and 2.4-fold higher in the baseline eGFR categories G2 and ≥ G3 compared to G1, but the baseline renal function was not found to be an independent predictor of AKI in our multiple regression model. Studies by Zhang et al. also failed to identify baseline renal function to be a risk factor for postoperative AKI after NSS [39, 49]. In contrast, our plots of natural cubic splines uniquely illustrated the association between IT and AKI risk with different effects depending on baseline renal function. These findings indicate that patients with baseline eGFR < 60 mL/min/1.73 m^2^ are at higher risk for AKI after NSS if renal ischaemia is applied.

Recently, Zhang et al. published a cohort study of 83 patients with solitary kidneys and showed that parenchymal mass reduction and IT are risk factors for postoperative AKI. Adjusting for parenchymal mass reduction, AKI was associated with 5–12% worse functional recovery depending on the AKI stage [39]. This is comparable to our results, although we could not identify AKI as a significant risk factor for long-term decreases in renal function after NSS. In our regression model, which was adjusted for tumour diameter as a surrogate parameter for removed renal parenchyma, we predicted a 0.2% relative reduction of eGFR at a median of 13 months postoperatively (IQR, 12–15) for every percentage of relative decrease of eGFR at a median of 1 day (IQR, 1–2) after NSS.

We detected a rate of new-onset CKD stage ≥3 of 27.9% in the overall cohort (NSS-C) until the latest recorded follow-up time. Our multiple regression model showed that baseline eGFR was the only significant predictor for CKD stage ≥3 at a median of 13 months (IQR, 12–15) after NSS, with an increasing risk of 11.0% for every baseline eGFR unit smaller. IT, ZI, AKI, or one of the two surgical approaches (LNSS-RI vs. ONSS-RI) were not significant prognostic factors for new-onset CKD stage ≥3.

The 27.9% rate of de novo CKD stage ≥3 in our study was comparable to previous data [25, 55, 56]. Clark et al. observed a postoperative rate of CKD stage ≥3 of 29.0% [55]. Muramaki et al. observed a rate of 39.4% [25], with no difference between LNSS and ONSS. Regression analysis predicting CKD-free survival or CKD development were not completely conformable, although both studies identified increasing age and lower baseline kidney function, but not IT, as independent risk factors. This was just recently confirmed by Lee et al. who showed that even prolonged warm IT was not associated with increased incidence of CKD [57]. However, the effect of ZI was not addressed in these studies.

Our study should be interpreted with consideration of its limitations. Retrospective data collection led to missing follow-up values, fairly large interquartile ranges for the measurement times C and E, and might have caused bias in the presented results. We were not able to investigate data regarding renal parenchyma preserved [39, 40, 49, 54] or tumour complexity described by a multimodal nephrometric score such as the RENAL [58] and PADUA [59] Nephrometry Score, or renal tumour contact surface area [60], which might have influenced the short-term change in renal function. Instead, we decided to use the tumour diameter, tumour location, and IT in the regression analyses as adjusting surrogate parameters for the amount of renal parenchyma removed. This strategy is supported by a study by Meyer et al., who used a precise three-dimensional volumetric analysis to prove that IT, tumour size, and endophytic/exophytic properties of a localised renal mass are the most important determinants of renal parenchymal volume loss [61]. However, AKI is currently stratified by increase in serum creatinine levels above baseline, with different classification schemes reporting different AKI rates (e.g., AKI criteria of KDIGO adopted for this study) resulting in higher AKI rates compared to the RIFLE (Risk, Injury, Failure, Loss of kidney function, and End-stage kidney disease) classification [62]. Moreover, this classical approach to define AKI does not take into account the reduction in nephron mass that occurs with NSS, and thus may overestimate the true incidence or grade of AKI [39]. Furthermore, changes in renal function measured by serum creatinine and eGFR might not be sufficiently accurate, especially when investigating patients with a normal contralateral kidney which compensates for loss of renal function. The MDRD equation we used is validated only until the age of 70 and was originally validated only for patients with CKD. Moreover, the MDRD equation categorised 7% more patients as having new-onset CKD after NSS compared to the CKD-EPI equation [56].

## Conclusions

Despite these limitations, the findings of this study suggest that AKI within 48 h postoperatively and CKD stage ≥3 develop in nearly 40.0 and 28.0% of patients after NSS for RCC, respectively. Baseline kidney function plays a key role in postoperative short-term and long-term relative changes in renal function, whereas renal ischaemia per se and longer IT negatively impact the short-term renal function and increase the risk for AKI. ONSS is significantly associated with an increased short-term impairment of renal function and increased risk for AKI. The tumour diameter and percentage change in renal function after a median of 1 day postoperatively appeared to constitute surrogate parameters to predict the percentage change in renal function after a median of 13 months postoperatively. The development of AKI was not directly associated with baseline renal function, but the impact of IT causes different dynamics in AKI rates depending on the baseline eGFR category. Otherwise, ZI surgery was not shown to influence long-term outcome variables significantly, which was recently confirmed by split renal scintigraphy [63].

Our findings are helpful for surgical planning, and they suggest either the application of a clampless NSS technique or at least the shortest possible IT to reduce the risk of short-term impairment of the renal function, which might prevent AKI, particularly regarding patients with baseline eGFR category ≥G3, and might reduce long-term impairment of renal function. The reason for the beneficial effects of LNSS on short-term renal function remains unclear, but different techniques of clamping of the renal artery (e.g., bulldog clamps in LNSS or Satinsky clamps in ONSS, like in or study), different renal/cortical reconstruction techniques, assuming tighter cortical renorrhaphy in ONSS [64], or a selection bias of higher tumour complexity in the open cases performed, may have influenced the worse short-term renal outcome observed in ONSS. Future investigations and strategies are needed to reduce ischaemia/reperfusion injury. Well-designed high quality prospective studies are needed to evaluate both the impact of nephrometric scores and renal ischemia/zero-ischemia on the renal functional outcomes in patients undergoing NSS for renal tumours (e.g. trial NTC02287987, [65]. Furthermore, studies evaluating renal function preservation after NSS should control for reconstructive renal injury. In addition, a modified definition of AKI in terms of surgery-related kidney injury that uses specific markers for renal tubular injury independently from a varying blood creatinine level, e.g. urinary biomarkers [66, 67], might help to better describe the postoperative short-term renal function and to predict the long-term renal function.

## Abbreviations

AKI: Acute Kidney Injury
AUA: American Urological Association
BMI: Body Mass Index
CKD: Chronic kidney disease
CKD-EPI: Chronic Kidney Disease Epidemiology Collaboration
CT: Computed Tomography
EAU: European Association of Urology
eGFR: Estimated Glomerular Filtration Rate
f: Female
IQR: Interquartile range
IT: Ischaemia time
KDIGO: Kidney Disease Improving Global Outcomes
LNSS: Laparoscopic nephron-sparing surgery
LNSS-RI: Laparoscopic nephron-sparing surgery group with intraoperative renal ischaemia
m: Male
MDRD: Modification of Diet in Renal Disease
MRI: Magnetic Resonance Imaging
n: Number
NSS ≥ G3: Nephron-sparing surgery group with baseline estimated glomerular filtration rate category ≥G3
NSS: Nephron-sparing surgery
NSS-AKI: Nephron-sparing surgery group with development of postoperative acute kidney injury
NSS-C: Overall nephron-sparing surgery cohort
NSS-G1: Nephron-sparing surgery group with baseline estimated glomerular filtration rate category G1
NSS-G2: Nephron-sparing surgery group with baseline estimated glomerular filtration rate category G2
NSS-NAKI: Nephron-sparing surgery group without development of postoperative acute kidney injury
NSS-NRI: Nephron-sparing surgery group without intraoperative renal ischaemia
NSS-RI: Nephron-sparing surgery group with intraoperative renal ischaemia
ONSS: Open nephron-sparing surgery
ONSS-RI: Open nephron-sparing surgery group with intraoperative renal ischaemia
OR: Odds Ratio
p.o.: Postoperative
RCC: Renal cell cancer
ref.: Reference
RIFLE: Risk, Injury, Failure, Loss of kidney function, and End-stage kidney disease
UICC: Union Internationale Contre le Cancer
ZI: Zero ischaemia
