# Correction to: Outcome of kidney function after ischaemic and zero-ischaemic laparoscopic and open nephron-sparing surgery for renal cell cancer

**DOI:** 10.1186/s12882-019-1264-7

**Published:** 2019-03-07

**Authors:** Jan Ebbing, Felix Menzel, Paolo Frumento, Kurt Miller, Bernhard Ralla, Tom Florian Fuller, Jonas Busch, Justin William Collins, Christofer Adding, Hans Helge Seifert, Peter Ardelt, Christian Wetterauer, Timm Westhoff, Carsten Kempkensteffen

**Affiliations:** 1 University Hospital Basel, Urological University Clinic Basel-Liestal, Spitalstrasse 21, 4051 Basel, Switzerland; 2 Department of Urology, Charité - University Hospital, Berlin, Germany; 3 Karolinska Institutet, Unit of Biostatistics, Institute of Environmental Medicine (IMM), Stockholm, Sweden; 4 Department of Molecular Medicine and Surgery (MMK), Karolinska Institutet, Stockholm, Sweden; 5 Marien Hospital Herne – University Clinic of the Ruhr-University Bochum, Medical Clinic I, Herne, Germany; 6 Department of Urology, Karolinska - University Hospital, Solna, Stockholm Sweden; 7 Department of Urology, Franziskus Hospital Berlin, Berlin, Germany

**Correction to: BMC Nephrology (2019) 20:40**

**https://doi.org/10.1186/s12882-019-1215-3**

Following publication of the original article [1], it was reported that Fig. 1i and Fig. 1j were omitted due to a typesetting mistake. In this Correction, the complete Fig. 1 is shown and the original publication of this article has been updated to correct this. The publisher apologises to the authors and readers for the inconvenience.

**Fig. 1:**
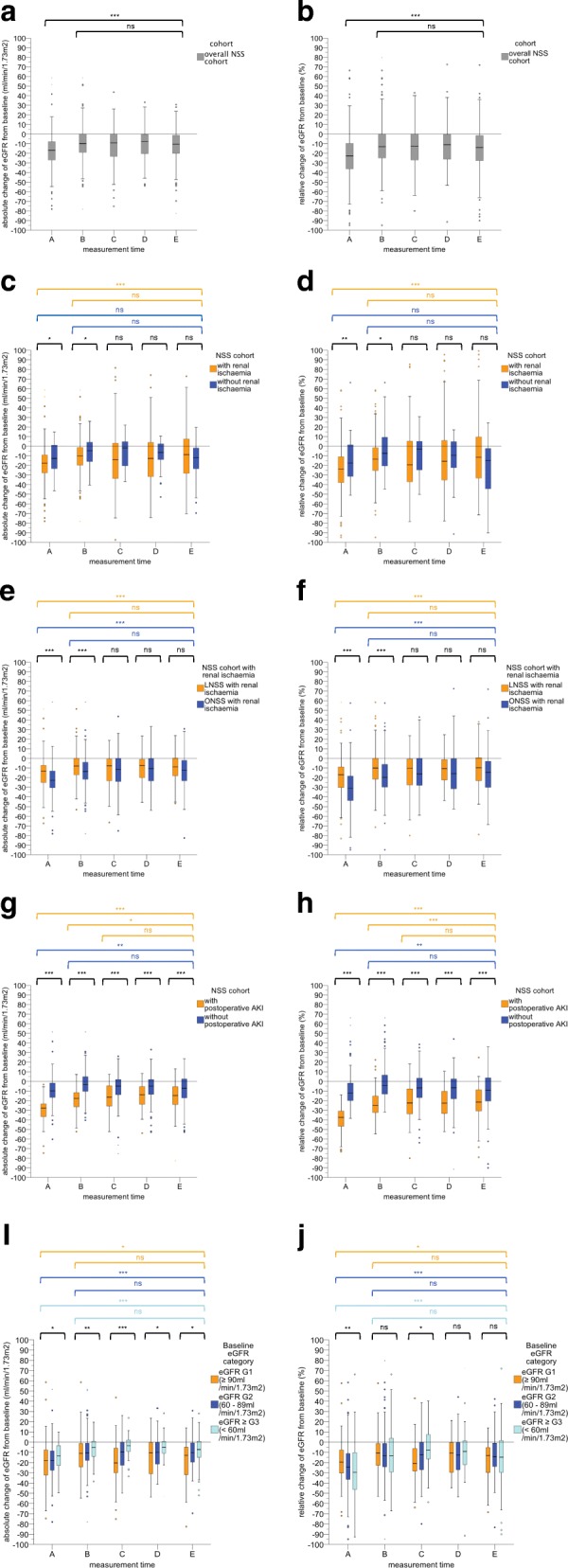
Box plots showing the postoperative course of the absolute (**a**/**c**/**e**/**g**/**i**) and relative (**b**/**d**/**f**/**h**/**j**) change (%) in eGFR at measurement times A-E for (**a**/**b**) the overall NSS cohort (NSS-C), (**c**/**d**) the NSS group with intraoperative renal ischaemia (NSS-RI) and without intraoperative renal ischaemia (NSS-NRI), (**e**/**f**) the LNSS group with intraoperative renal ischaemia (LNSS-RI), the ONSS group with intraoperative renal ischaemia (ONSS-RI), (**g**/**h**) the NSS group with postoperative AKI (NSS-AKI), the NSS group without postoperative AKI (NSS-NAKI), and (**i**/**j**) NSS group with a baseline eGFR category G1 (NSS-G1), NSS group with a baseline eGFR category G2 (NSS-G2), and NSS group with a baseline eGFR category ≥G3 (NSS ≥ G3). Definition of measurement times **a**-**e**: (**a**) highest change in eGFR from baseline during the planned hospital stay at a median of 1 day postoperatively (IQR, 1–2), (**b**) change in eGFR from baseline prior to discharge from hospital at a median of 4 days postoperatively (IQR, 2–6), (**c**) change in eGFR from baseline at a median of 47 days postoperatively (IQR, 30–105), (**d**) a median of 13 months postoperatively (IQR, 12–15), and (**e**) a median of 50 months postoperatively (IQR, 35–81). Asterisks indicate significant changes from baseline in the level of absolute and relative changes in eGFR over the course of the observation period (Friedman’s test as a post hoc pairwise multiple comparison test) or between the compared groups at each measurement time (non-parametric Mann-Whitney U test). * *p* < 0.05, ** *p* < 0.01, *** *p* < 0.001, (ns) not significant. eGFR, estimated glomerular filtration rate; NSS, nephron-sparing surgery; LNSS, laparoscopic nephron-sparing surgery; ONSS, open nephronsparing surgery; AKI, acute kidney injury; IQR, interquartile range

Additionally, the authors reported that the caption of Table 4 was incorrectly presented as “Multiple linear regression analysis”. The correct presentation of this table caption is “Multiple regression analysis”. And Table 4 with its corrected caption can be found on page 3-4.

**Table 4:** Multiple regression analysis

a (model 1)	Regression coefficient β	Multiple linear regression 95% CI	*p*-value
Baseline eGFR (mL/ml/1.73 m^22^)	- 0.20	- 0.38 - − 0.02	0.03
Baseline Haemoglobin (mg/dL)	0.51	- 0.86 - 1.89	0.46
Tumour diameter (cm)	0.67	- 0.43 - 1.76	0.24
Tumour locus central (ref.) vs. peripheral	0.43	- 3.80 - 4.67	0.84
Surgical approach LNSS (ref.) vs. ONSS	- 13.48	- 17.65 - − 9.32	< 0.001
Sex male (ref.) vs. female	- 3.28	- 7.80 - 1.25	0.16
Age (years)	- 0.17	- 0.36 - 0.01	0.06
BMI (kg/m^2^)	- 0.88	- 1.36 - − 0.41	< 0.001
Hypertension no (ref.) vs. yes	- 0.78	- 4.75 - 3.18	0.70
Ischaemia time (min)	- 0.27	- 0.41 - − 0.13	< 0.001
Operative time (min)	- 0.06	- 0.09 - − 0.03	< 0.001
Preoperative ureter stenting no (ref.) vs. yes	- 0.46	- 5.64 - 4.71	0.86
Intraoperative blood transfusions no (ref.) vs. yes	- 3.29	- 11.91 - 5.33	0.45
Postoperative complications no (ref.) vs. yes	- 3.36	- 8.42 - 1.70	0.19
Clavien-Dindo score < 3 (ref.) vs. ≥ 3	- 10.98	- 18.47 - − 3.48	0.004
b (model 2)	Regression coefficient β	Multiple linear regression 95% CI	*p*-value
Baseline eGFR (mL/ml/1.73 m^22^)	- 0.29	- 0.49 - − 0.09	0.005
Baseline Haemoglobin (mg/dL)	- 0.32	- 1.95 - 1.31	0.70
Relative change of eGFR from baseline at time A (%)	0.18	0.03 - 0.33	0.02
AKI 48 h p.o. no (ref.) vs. yes	- 2.11	- 9.01 - 4.79	0.55
Tumour diameter (cm)	- 1.76	- 2.87 - − 0.66	0.002
Tumour locus central (ref.) vs. peripheral	- 0.30	- 5.14 - 4.54	0.90
Surgical approach LNSS (ref.) vs. ONSS	1.13	- 4.17 - − 6.44	0.67
Sex male (ref.) vs. female	1.63	- 3.43 - 6.70	0.53
Age (years)	- 0.10	- 0.33 - 0.13	0.40
BMI (kg/m2)	0.15	- 0.39 - 0.70	0.58
Hypertension no (ref.) vs. yes	- 2.11	- 6.82 - 2.60	0.38
Ischaemia time (min)	0.03	- 0.14 - 0.21	0.72
Operative time (min)	0.01	- 0.03 - 0.05	0.54
Preoperative ureter stenting no (ref.) vs. yes	- 4.35	- 10.46 - 1.77	0.16
Intraoperative blood transfusions no (ref.) vs. yes	4.12	- 6.29 - 14.52	0.44
Postoperative complications no (ref.) vs. yes	1.20	- 4.77 - 7.18	0.69
Clavien-Dindo score < 3 (ref.) vs. ≥ 3	4.43	- 4.28 - 13.14	0.32
c (model 3)	OR	Multiple logistic regression 95% CI	*p*-value
Baseline eGFR (mL/ml/1.73 m^22^)	0.99	0.96 - 1.01	0.30
Baseline Haemoglobin (mg/dl)	0.85	0.70 - 1.03	0.10
Tumour diameter (cm)	0.94	0.81 - 1.08	0.35
Tumour locus central (ref.) vs. peripheral	1.20	0.70 - 2.05	0.51
Surgical approach LNSS (ref.) vs. ONSS	3.87	2.17 - 6.92	< 0.001
Sex male (ref.) vs. female	2.51	1.35 - 4.67	0.004
Age (years)	1.01	0.99 - 1.04	0.26
BMI (kg/m^2^)	1.13	1.06 - 1.21	< 0.001
Hypertension no (ref.) vs. yes	1.05	0.63 - 1.74	0.85
Ischaemia time (min)	1.02	1.00 - 1.04	0.046
Operative time (min)	1.01	1.00 - 1.01	0.002
Preoperative ureter stenting no (ref.) vs. yes	0.92	0.46 - 1.83	0.81
Intraoperative blood transfusions no (ref.) vs. yes	0.73	0.22 - 2.45	0.61
Postoperative complications no (ref.) vs. yes	1.79	0.92 - 3.48	0.08
Clavien-Dindo score < 3 (ref.) vs. ≥ 3	2.14	0.68 - 6.72	0.19
d (model 4)	OR	Multiple logistic regression 95% CI	*p*-value
Baseline eGFR (mL/ml/1.73 m^2^)	0.89	0.85 - 0.92	< 0.001
Baseline Haemoglobin (mg/dL)	0.99	0.73 - 1.35	0.95
Relative change of eGFR from baseline at time A (%)	0.98	0.98 - 1.01	0.12
AKI 48 h p.o. no (ref.) vs. yes	1.23	0.39 - 3.85	0.72
Tumour diameter (cm)	0.93	0.71 - 1.21	0.58
Tumour locus central (ref.) vs. peripheral	1.35	0.56 - 3.15	0.49
Surgical approach LNSS (ref.) vs. ONSS	1.69	0.67 - 4.24	0.26
Sex male (ref.) vs. female	0.63	0.24 - 1.67	0.35
Age (years)	0.99	0.95 - 1.04	0.75
BMI (kg/m^2^)	0.97	0.87 - 1.07	0.50
Hypertension no (ref.) vs. yes	1.62	0.66 - 4.00	0.29
Ischaemia time (min)	1.01	0.98 - 1.04	0.55
Operative time (min)	1.00	0.99 - 1.01	0.86
Preoperative ureter stenting no (ref.) vs. yes	1.26	0.41 - 3.86	0.68
Intraoperative blood transfusions no (ref.) vs. yes	0.95	0.08 - 11.05	0.97
Postoperative complications no (ref.) vs. yes	0.67	0.22 - 2.00	0.47
Clavien-Dindo score < 3 (ref.) vs. ≥ 3	1.37	0.22 - 8.41	0.73

Multiple linear regression analysis for models 1 and 2 including ischaemia time as a continuous variable investigating predictors of the relative change (%) of eGFR from baseline at (a) measurement time A (median, 1 day p.o.; IQR, 1–2) and at (b) at measurement time D (median, 13 months p.o.; IQR 12–15), and multiple logistic regression analysis for models 3 and 4 including ischaemia time as a continuous variable investigating (c) predictors for the development of postoperative AKI within 48 h p.o. and (d) predictors for the development of postoperative new-onset CKD stage ≥ 3 (eGFR < 60 mL/min/1.73 m2) within measurement time D

The regression models are based on pooled estimates from 100 imputed datasets. A *p*-value < 0.05 is regarded as statistically significant

*eGFR* estimated glomerular filtration rate, *CKD* chronic kidney disease, *AKI* acute kidney injury, *LNSS* laparoscopic nephron-sparing surgery, *ONSS* open nephron-sparing surgery, *BMI* body mass index, *OR* odds ratio
